# Global Subjective Assessment and Mini Nutritional Assessment Short Form Better Predict Mortality Than GLIM Malnutrition Criteria in Elderly Patients with Hip Fracture

**DOI:** 10.3390/nu15081828

**Published:** 2023-04-10

**Authors:** Francisco José Sánchez-Torralvo, Verónica Pérez-del-Río, María García-Olivares, Nuria Porras, Jose Abuín-Fernández, Manuel Francisco Bravo-Bardají, David García-de-Quevedo, Gabriel Olveira

**Affiliations:** 1 Unidad de Gestión Clínica de Endocrinología y Nutrición, Hospital Regional Universitario de Málaga, 29007 Malaga, Spain; 2 Instituto de Investigación Biomédica de Málaga (IBIMA), Plataforma Bionand, 29010 Malaga, Spain; 3 Departamento de Medicina y Dermatología, Facultad de Medicina, University of Malaga, 29010 Malaga, Spain; 4 Unidad de Gestión Clínica de Cirugía Ortopédica y Traumatología, Hospital Regional Universitario de Málaga, 29010 Malaga, Spain; 5 Centro de Investigación Biomédica en Red de Diabetes y Enfermedades Metabólicas Asociadas (CIBERDEM), Instituto de Salud Carlos III, 28029 Madrid, Spain

**Keywords:** hip fracture, elderly, malnutrition, Mini Nutritional Assessment Short Form, Subjective Global Assessment, GLIM criteria

## Abstract

The objective of our study is to determine the prevalence of malnutrition in elderly patients with fragility hip fractures through different diagnostic tools and to determine which nutritional assessment tool better predicts mortality. Methods: This is a prospective study in patients over 65 years of age hospitalized with a diagnosis of hip fracture. A nutritional assessment was performed using several tools: the Mini Nutritional Assessment Short Form (MNA-SF), the Subjective Global Assessment (SGA), and the GLIM criteria. For the definition of low muscle mass, four different methods were used: hand grip strength (HGS), calf circumference (CC), anthropometry, and bioelectrical impedance (BIA). Mortality was registered at three, six and twelve months. Results: 300 patients were included, 79.3% female, mean age 82.9 ± 7.1 years. The MNA-SF found 42% at risk of malnutrition, and 37.3% malnourished. Using SGA, there were 44% with moderate malnutrition, and 21.7% with severe malnutrition. In application of the GLIM criteria, 84.3%, 47%, 46%, and 72.7% of patients were malnourished when HGS, anthropometry, BIA, and CC were used, respectively. Mortality was 10%, 16.3% and 22% at 3, 6 and 12 months, respectively. In malnourished patients according to MNA-SF, mortality was 5.7 times greater [95%CI 1.3–25.4; *p* = 0.022] at 6 months and 3.8 times greater [95%CI 1.3–11.6; *p* = 0.018] at 12 months. In malnourished patients according to SGA, mortality was 3.6 times greater [95%CI 1.02–13.04; *p* = 0.047] at 3 months, 3.4 times greater [95%CI 1.3–8.6; *p* = 0.012] at 6 months and 3 times greater [95%CI 1.35–6.7; *p* = 0.007] at 12 months. Conclusion: The prevalence of malnutrition in patients admitted for fragility hip fracture is high. The SGA and MNA-SF are postulated as adequate tools to diagnose malnutrition in these patients, with predictive value for mortality at three, six, and twelve months.

## 1. Introduction

Given the current aging of the population, there is an increasing incidence of osteoporosis. This has led to increased interest in the prevention and treatment of fragility fractures, which are those produced by low impact (such as a fall from a height corresponding to a standing position, mainly in the humerus, wrist, vertebrae, and hip) [1]. Specifically, hip fracture is the most important, due to its high risk of mortality and refracture, which also entails a large economic cost [2,3]. The global incidence of hip fracture stands at 1.7 million cases per year worldwide [4], of which around 620,000 are in Europe [5]. Since 2011, evidence has emerged of the usefulness of the existence of Fracture Coordination Units (UCF or FLS, Fracture Liaison Services), which focus their activity on the secondary prevention of fragility fractures [6]. These units evaluate in a multidisciplinary way various aspects of the process of secondary prevention of fractures [7] and application of its methods has shown a significant reduction of all-cause mortality [8].

In this context, the nutritional aspect of these patients is very important, since there is a positive association between the presence of malnutrition and the rate of hip fractures [9]. Current data shows great variability in the prevalence of malnutrition, probably due to the existence of non-standardized criteria. However, most of the existing literature refers to an approximate prevalence of 20–30% malnutrition and a 40–50% risk of malnutrition [9,10,11,12,13,14]. Other studies show higher figures [15]. Malnutrition relates to complications, lower functional recovery, and higher mortality. There is also an association between morbidity and mortality and nutritional status [9,12,16], although this association is not as established as it is with other pathologies closely related to malnutrition, such as oncological pathology [17]. A correct nutritional intervention in these patients can prevent complications [18] and, in addition, could reduce recovery times and mortality after the intervention [19,20].

The Global Leadership Initiative on Malnutrition (GLIM) criteria for malnutrition were introduced in 2018 [21], but to our knowledge few studies have included them in the assessment of the nutritional status of patients admitted for hip fracture [22,23]. Although some studies have explored the relationship between malnutrition and mortality in these patients [14,24,25], to date, we are not aware of studies that have used GLIM to verify the relationship between malnutrition and mortality, nor studies that compare the results of the application of different diagnostic tools for malnutrition.

Our hypothesis is that the prevalence of malnutrition in patients admitted for fragility hip fracture in the Trauma Unit could be high and be related to an increase in mortality.

The objective of our study is to determine the prevalence of malnutrition in elderly patients with fragility hip fracture through different diagnostic tools and to determine which nutritional assessment tool better predicts mortality at 3, 6, and 12 months.

## 2. Materials and Methods

This is a prospective study, in patients over 65 years of age hospitalized with a diagnosis of hip fracture in the Trauma Surgery Unit of the Regional Hospital of Malaga, between September 2019 and February 2021. Fracture type and the presence of a previous fracture were recorded. Medical comorbidities were measured by the Charlson Comorbidity Index (CCI) [26]. Pre-fracture functional status was assessed by means of the Barthel index [27] and the Functional Ambulation Category Scale (FAC) [28]. Analytical data for C-reactive protein and albumin were collected and the CRP/albumin ratio was calculated.

A nutritional assessment in the first 24–48 h after the intervention was performed. This assessment was carried out using several tools:

- the Mini Nutritional Assessment Short Form (MNA-SF) [29], replacing the body mass index (BMI) item with calf circumference (CC).
- the Subjective Global Assessment (SGA) [30], and
- the Global Leadership in Malnutrition (GLIM) criteria for diagnosis of malnutrition [21].

Height was calculated with a stadiometer (Holtain Limited, Crymych, UK) when possible and weight was calculated fasting with a scale set to 0.1 kg (SECA 665, Hamburg, Germany). When the determination of height and weight was not possible, data reported by the patient were used.

### 2.1. Malnutrition according to the GLIM Criteria

To diagnose malnutrition according to the GLIM criteria, at least one phenotypic criterion and one etiological criterion must be present [21]. All patients were considered to have at least one etiological criterion, due to the existence of an inflammatory response after having undergone surgery for a hip fracture in the previous days. This was confirmed by the CRP/albumin ratio.

The following phenotypic criteria were evaluated: unintentional weight loss (> 5% in 6 months), low BMI (for age < 70 years, a BMI ≥ 20 kg/m^2^ was considered normal; for age ≥ 70, a BMI ≥ 22 kg/m^2^ was established as normal), and/or reduction in muscle mass. For the definition of low muscle mass, four different methods were used: a low hand grip strength (represented by the fifth percentile population) [31], a low calf circumference (CC), or a low fat-free mass index (FFMI) according to ESPEN cut-off points [32], this being determined by anthropometry (triceps skinfold) and bioelectrical impedance (BIA).

BIA was performed with the Akern BIA-101/Nutrilab analyzer (Akern SRL, Pontassieve, 160 Florence, Italy). Measurements were taken in the supine position, with the upper (30°) and lower (45°) limbs abducted. Software (AKERN Bodygram Dashboard, Pontassieve, Florence, Italy) was used to determine the FFMI.

Measurement of the triceps skinfold was performed using a Holtain caliper (Holtain Limited). Measurements were taken in triplicate in the dominant arm and the mean was calculated. The percentages and kilograms of fat mass and fat-free mass (FFM) were estimated according to the Siri and Durnin and Womersley formulas [33,34]. For the FFMI, the cut-off points established by ESPEN were applied, considering low muscle mass for values <15 kg/m^2^ in women and <17 kg/m^2^ in men [32].

Calf circumference (CC) was measured using a non-elastic tape at the point of the greatest circumference. A low CC was defined using the cutoff points suggested in the GLIM criteria guidelines: 33 cm for men and 32 cm for women [35].

Hand grip strength was measured in the dominant hand with a Jamar dynamometer (Asimow Engineering Co., Los Angeles, CA, USA). The patients performed the test with the shoulder adducted and the forearm in neutral rotation, the elbow flexed to 90°, and the forearm and wrist in a neutral position. Patients were asked to perform three consecutive contractions one minute apart, and the mean value was calculated. Results were expressed in absolute terms, and scores below the fifth percentile of the population were considered to have low hand grip strength. [31].

### 2.2. Follow-Up

After discharge, a telematic follow-up was carried out (through a review of the clinical history) of the evolution of the patients, recording mortality at three, six and twelve months.

### 2.3. Statistical Analysis

Quantitative variables were expressed as mean ± standard deviation. The relationship between malnutrition diagnosis using different tools and mortality was estimated using the chi-square test, with Fisher’s correction when necessary. For the concordance between diagnostic techniques, the kappa coefficient was used. The variables that showed an association with mortality in the chi-square test were included in a multivariate logistic regression model to assess the association between mortality and malnutrition, controlling for confounding variables such as sex, age and Charlson Comorbidity Index. For calculations, significance was set at *p* < 0.05 for two tails. Data analysis was performed using the SPSS 26.0 program (SPSS Inc., Chicago, IL, USA).

## 3. Results

**Figure 1:**
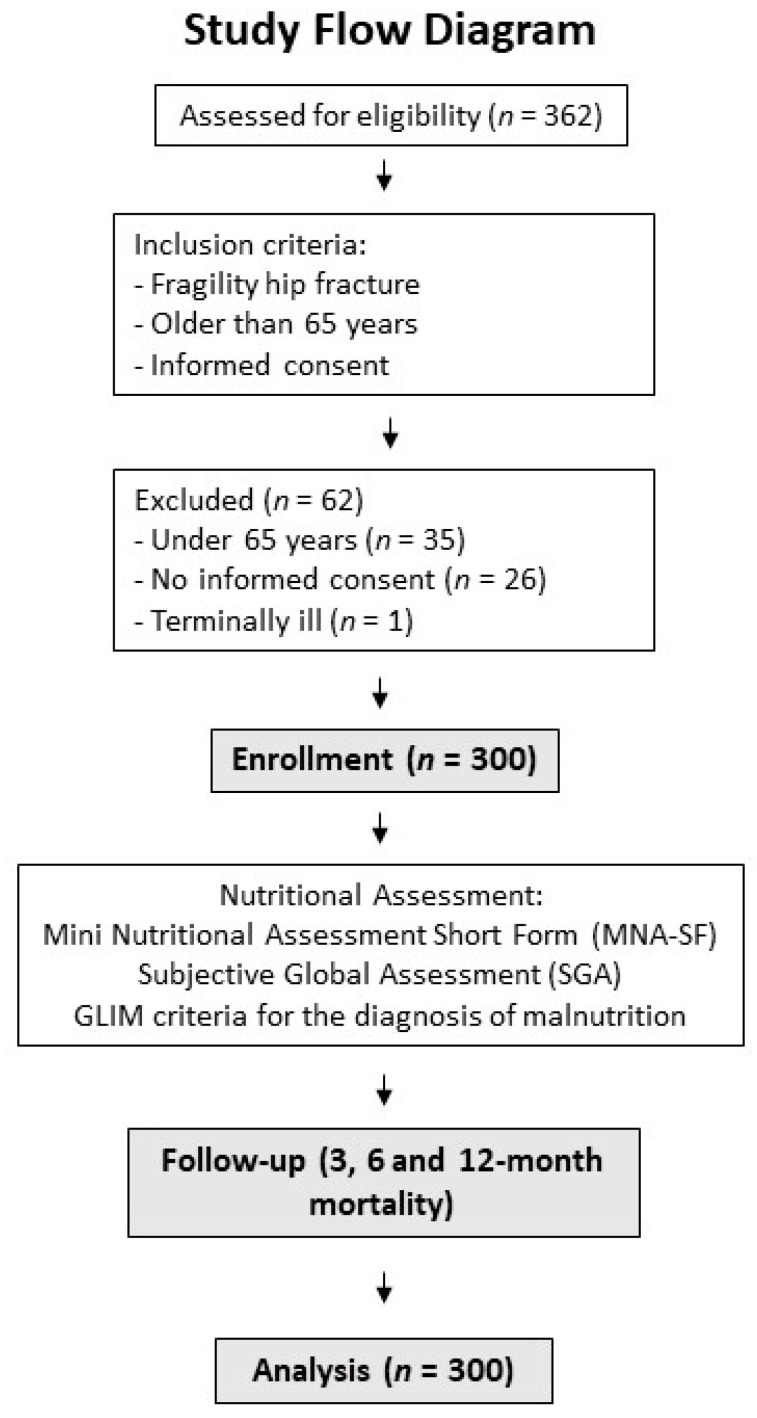
Study flow diagram and research methodology.

A total of 300 patients were included (Figure 1), 62 men (20.7%) and 238 women (79.3%), with a mean age of 82.9 ± 7.1 years. The estimated mean BMI was 25.8 ± 5.1 kg/m^2^.

The general characteristics of the sample are shown in Table 1.

The mean triceps skinfold was 11.9 ± 4 mm for men, giving an FFMI by anthropometry of 19.4 ± 8.6 kg/m^2^ (25.8% below 17 kg/m^2^). In women, the mean triceps skinfold was 15.7 ± 6.1 mm, determining an FFMI of 17.4 ± 2.9 kg/m^2^ (19.7% below 15 kg/m^2^).

The mean calf circumference was 32.4 ± 2.8 cm in men (54.8% below 33 cm) and 30.7 ± 3.8 cm in women (67.2% below 32 cm).

The FFMI by BIA was 20.9 ± 9.6 kg/m^2^ for men (8.7% below 17 kg/m^2^) and 17.5 ± 2.1 kg/m^2^ for women (9.1% below 15 kg/m^2^).

HGS showed a mean of 19.7 ± 9.7 kg for men (69.4% below the p5 population percentile) and 7.7 ± 6.4 kg for women (72.3% below the population percentile p5). Body composition parameters are shown in Table 2.

**Figure 2:**
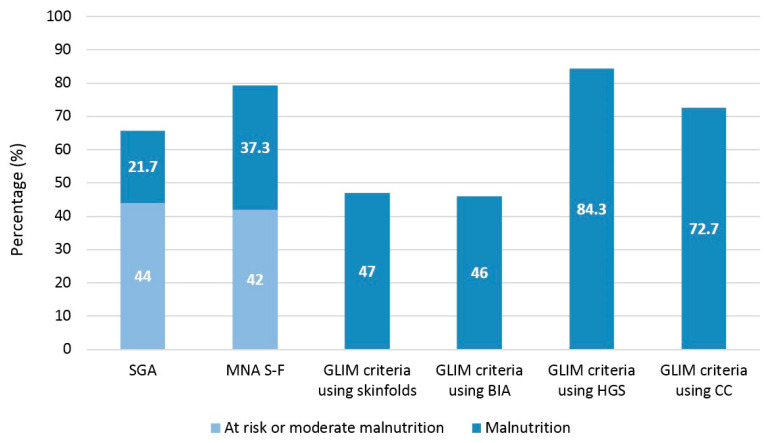
Malnutrition diagnosis according to the tool used. SGA: Subjective Global Assessment; MNA S-F: Mini Nutritional Assessment Short Form; GLIM: Global Leadership on Malnutrition; BIA: Bioelectrical impedance analysis; HGS: Handgrip Strength; CC: calf circumference.

Regarding the prevalence of malnutrition (Figure 2), the MNA-SF found 20.7% normally nourished, 42% at risk of malnutrition, and 37.3% malnourished. Using SGA, 34.3% were found to be normally nourished, 44% with moderate malnutrition, and 21.7% with severe malnutrition (kappa coefficient of 0.53 with MNA-SF; *p* < 0.001).

In application of the GLIM criteria, 68 patients (22.7%) presented a low BMI and 113 (37.7%) a loss of more than 5% of body weight in the previous months. Considering the previous phenotypic criteria and using HGS as a determinant of muscle mass, we found 84.3% of patients undernourished; 47% when anthropometry was used, 46% when BIA was used, and 72.7% when CC was used (kappa coefficient of 0.39, 0.37, 0.41, 0.37 and with SGA respectively; *p* < 0.001). We found good agreement between GLIM with anthropometry and GLIM with anthropometry (kappa coefficient of 0.94; *p* < 0.001).

During follow-up, a total of 30 patients (10%) died in the first 3 months after the intervention, 49 patients (16.3%) at 6 months, and 66 patients (22%) at 12 months.

Table 3 shows the results of the analysis that relates 3-, 6- and 12-months mortality to the diagnosis of malnutrition according to the various nutritional assessment tools.

An association was found between age and the Charlson Comorbidity Index, and mortality at 3, 6, and 12 months (*p* < 0.001 at all times). For this reason, these variables were included in the logistic regression adjustment.

Malnutrition according to GLIM using HGS was not included in the regression, since the absence of positive events in the few normally nourished patients prevented a correct risk analysis. Table 4 shows the relationship between mortality at 3, 6 and 12 months, and the diagnosis of malnutrition using SGA and MNA SF, adjusted for age, sex and Charlson Comorbidity Index.

## 4. Discussion

In our study, the prevalence of malnutrition in elderly patients operated on for fragility hip fracture is high, hovering between 45 and 85% depending on the nutritional assessment tool used. These figures agree with those previously presented by other authors [9,10,11,12,13,15,20].

Previously, the most commonly used diagnostic tool for the diagnosis of malnutrition has been the MNA-SF [9,11,12,13,15,20,25]. The use of MNA-SF as a tool for diagnosing malnutrition is supported by its ease of application and reproducibility, without the need for biochemical determinations. Although the “Short Form” version was designed as a screening test, it can also be used for nutritional assessment [29]. Its use is widespread in the geriatric population. In our work, we used calf circumference instead of BMI, since the exact weight could not be available in some cases. As an alternative to the MNA-SF, the use of SGA in the nutritional assessment of hospitalized patients is justified since it is a valid, sensitive tool with prognostic value and adequate concordance with other tools [36,37].

In our sample, mortality at 3, 6 and 12 months presented a risk up to 3–4 times higher in patients who were malnourished according to SGA than those who were normo-nourished. These data are consistent with those of the study of Miu et al., in which hospital mortality was higher in malnourished individuals compared to patients at risk of malnutrition and normally nourished patients, presenting this trend also at 6 months, although without reaching statistical significance [9]. The authors postulated that there could be certain limitations in the MNA for mortality prediction, such as the use of BMI or the absence of analytical parameters. This, however, differs from our results, in which malnourished patients according to MNA-SF presented a risk of mortality between 3 and 6 times higher than normally nourished patients. In this case, the different results could be justified by our decision to use the CC instead of the BMI when applying the MNA-SF.

Hand grip strength is a technique that correlates very well with lean mass and is an inexpensive tool that is easy to reproduce [31]. The prevalence of low hand grip strength values is very high in our sample, something that was described in similar populations, reaching over 90% [10,38]. In previous studies carried out on patients admitted to our hospital, we already found a high prevalence of low hand grip strength [38]. In the present study, patients with a recent hip intervention were included, so that in most of the cases, greater difficulty in sitting could determine lower values. A poor technique could have implied an artificially high prevalence of low hand grip strength, which could lead to an overestimation of malnourished patients when applying the GLIM criteria, something that has had a direct impact on the estimation of its association with mortality in the statistical analyses of our study. For this reason, HGS does not seem to be a reliable tool for these patients.

In recent years, the use of bioelectrical impedance analysis in nutritional assessment has spread. Some authors have included BIA in the assessment of elderly patients operated on for a hip fracture [10,22,38], including muscle mass parameters, such as the musculoskeletal index (SMI), although presenting disparate data. In our study, the FFMI was used as a determinant of muscle mass, presenting low values in 8–9% of the patients, which could be interpreted as an overestimation of muscle mass by the BIA. Nevertheless, a recent study has investigated the use of the GLIM criteria for malnutrition in patients with hip fractures, using BIA as determinant of muscle mass, determining that is useful for predicting gait ability at discharge during acute hospitalization [22].

The use of calf circumference in patients with hip fracture is common in estimating muscle mass [9,23]. In a recent study, CC was found to be a valuable tool in predicting sarcopenia risk compared with other screening tools [39]. Our study determined a low CC according to the cut-off points recommended in the GLIM criteria guidelines (33 cm for men and 32 cm for women) while, for applying the MNA-SF, a single cut-off point is used at 31 cm. With a lower cut-off point, the MNA-SF detected a lower percentage of patients with low muscle mass, but both the prevalence of malnutrition and the relationship with mortality were higher than when applying the GLIM criteria, possibly due to the use of other subjective parameters.

To date, only two studies have applied the GLIM criteria for the diagnosis of malnutrition in patients with fragility hip fractures [22,23]. In a retrospective Swedish study [23], phenotypic criteria were assessed, using calf circumference as a determinant of muscle mass, although the prevalence of malnutrition was not detailed. On the other hand, the study by Kobayashi et al. used the BIA as a determinant of muscle mass and found a prevalence of malnutrition of 73.9%. The fundamental difference to our study was the use of the skeletal muscle mass index (SMI) instead of the FFMI. In addition, mortality was not studied and non-weight bearing patients were excluded [22].

Although the prevalence of malnutrition determined using anthropometry and BIA in the application of the GLIM criteria was similar in our sample (good concordance), the low predictive value of mortality could place the use of the GLIM criteria one step below SGA and MNA-SF in this group of patients.

On the other hand, although the use of hand grip strength in the application of the GLIM criteria could have a good prognostic value for mortality according to our results [37], the great discrepancy found in the results, motivated by its difficulty in performance after a hip intervention, makes its use as a determinant of muscle mass in this case not recommended.

It is worth noting the greater concordance found in our study between SGA and MNA S-F than between SGA and the GLIM criteria, regardless of the technique used to measure muscle mass. This may be due to the fact that the GLIM criteria use BMI as one of sources of the phenotypic data, and in the case of our patients, this data was in most cases reported verbally. On the other hand, the difficulty in measuring muscle mass with the techniques used could also have led to a greater disparity in the estimation of the prevalence of malnutrition.

Based on other previously published studies [9,10,11,12,13,18,19,40], the implementation of a generalized nutritional screening for those patients with fragility hip fractures could reduce the incidence of refractures in the case of carrying out an appropriate nutritional intervention, as well as a possible reduction in the average stay and in complications. In our study, we have not evaluated a nutritional intervention, but our results indicate that the application of a systematic nutritional screening and assessment protocol to all those patients admitted for hip fragility fracture could be useful for the early detection of subjects at risk or malnourished.

Our study has several strengths. It is a prospective study with a large number of subjects and medium-term follow-up. In addition, it uses simple techniques for the measurement and definition of muscle mass loss and the presence of malnutrition, something that can be useful when other methods are not available.

In turn, there are several potential limitations. This is a single-center observational study, so the results need to be interpreted in the appropriate population context, particularly in populations with different surgical approaches to hip fracture, and no causal relationships can be established. On the other hand, patients underwent hip surgery in the hours before the assessment, so the results of some diagnostic techniques, such as hand grip strength and BIA, could be affected. In most cases, patients’ height and weight were reported verbally due to their inability to stand. This can affect the calculations of techniques such as BIA. For this reason, we recommend using calf circumference instead of BMI when using the MNA-SF if weight and height cannot be measured correctly.

## 5. Conclusions

In conclusion, the prevalence of malnutrition in elderly patients admitted for fragility hip fracture is high. The SGA and MNA-SF are postulated as adequate tools for the diagnosis of malnutrition, with predictive value for mortality at 3, 6 and 12 months in elderly patients operated on for fragility hip fracture. Further studies are needed to analyze the role of the GLIM criteria in diagnosing malnutrition in these patients.

## Figures and Tables

**Table 1:** General features.

		*n* = 300
Age (years)	mean ± SD	82.9 ± 7.1
Sex	*n* (%)	
Men		62 (20.7)
Women		238 (79.3)
Charlson Comorbidity Index	mean ± SD	5.67 ± 1.91
Barthel Index	mean ± SD	73.71 ± 27.72
Functional Ambulation Category Scale	*n* (%)	
0		78 (26.1)
1,2,3		206 (68.6)
4,5		16 (5.3)
Type of fracture	*n* (%)	
Pertrochanteric		135 (45)
Sub-capital		131 (43.7)
Sub-tronchanteric		18 (6)
Basi-cervical		15 (5)
Transcervical		1 (0.3)
Previous fracture	*n* (%)	34 (11.3)
C-reactive protein (CRP) (mg/l)	mean ± SD	115.4 ± 56.5
Albumin (g/dL)	mean ± SD	2.5 ± 0.4
CRP/Albumin ratio	mean ± SD	47.1 ± 25.5
Length of stay	mean ± SD	8.1 ± 5.4
3-month exitus	*n* (%)	30 (10)
6-month exitus	*n* (%)	49 (16.3)
12-month exitus	*n* (%)	66 (22)

Abbreviations: BMI = Body Mass Index; m = mean; SD = Standard Deviation.

**Table 2:** Body composition parameters.

		*n* = 300
BMI (kg/m^2^)	mean ± SD	
Men		25.9 ± 3.5
Women		25.8 ± 5.4
Triceps skinfold (mm)	mean ± SD	
Men		11.9 ± 4
Women		15.7 ± 6.1
Calf circumference (cm)	mean ± SD	
Men		32.4 ± 2.8
Women		30.7 ± 3.8
Fat-free mass (anthropometry) (kg)	mean ± SD	
Men		53.4 ± 8.3
Women		42.8 ± 7.3
FFMI (anthropometry) (kg/m^2^)	mean ± SD	
Men		19.4 ± 8.6
Women		17.5 ± 2.1
Phase angle (°)	mean ± SD	
Men		5.18 ± 1.13
Women		4.5 ± 0.94
Fat-free mass (BIA) (kg)	mean ± SD	
Men		57.6 ± 7.8
Women		42.9 ± 5.4
FFMI (BIA) (kg/m^2^)	mean ± SD	
Men		20.9 ± 9.6
Women		15.4 ± 1.5
Handgrip strength (kg)	mean ± SD	
Men		19.7 ± 9.7
Women		7.7 ± 6.4

BMI: body mass index; SD: standard deviation; FFMI: fat-free mass index; BIA: Bioelectrical impedance analysis.

**Table 3:** Relationship between malnutrition and mortality.

	3-Month Mortality			6-Month Mortality			12-Month Mortality		
	Malnourished	Normo-Nourished	*p* Value	Malnourished	Normo-Nourished	*p* Value	Malnourished	Normo-Nourished	*p* Value
Malnutrition according to SGA	23 (13.7%)	3 (2.9%)	0.002	43 (21.8%)	6 (5.8%)	<0.001	57 (29.9%)	9 (8.7%)	<0.001
Malnutrition according to MNA-SF	29 (12.2%)	1 (1.6%)	0.006	47 (19.7%)	2 (3.2%)	0.001	62 (26%)	4 (6.5%)	<0.001
Malnutrition according to GLIM using skinfolds	13 (9.2%)	17 (10.7%)	0.41	27 (19.1%)	22 (13.8%)	0.139	35 (24.8%)	31 (19.5%)	0.166
Malnutrition according to GLIM using BIA	11 (8.3%)	13 (7.7%)	0.527	26 (19%)	18 (11.3%)	0.056	32 (24.8%)	26 (16.2%)	0.057
Malnutrition according to GLIM using HGS	30 (11.8%)	0 (0%)	0.005	49 (19.4%)	0 (0%)	<0.001	65 (25.7%)	1 (2.1%)	<0.001
Malnutrition according to GLIM using CC	23 (10.6%)	7 (8.5%)	0.391	39 (17.9%)	10 (12.2%)	0.155	53 (24.3%)	13 (15.9%)	0.076

SGA: Subjective Global Assessment; MNA SF: Mini Nutritional Assessment Short Form; GLIM: Global Leadership on Malnutrition; BIA: Bioelectrical impedance analysis; HGS: Handgrip Strength; CC: calf circumference.

**Table 4:** Relationship between mortality and malnutrition according to SGA and MNA SF, adjusted for age, sex and Charlson Comorbidity Index.

	Crude				Adjusted			
	Odds Ratio	95% CI		*p* Value	Odds Ratio	95% CI		*p* Value
		Lower	Upper			Lower	Upper	
Malnutrition according to SGA								
3-month mortality	5.29	1.57	17.89	0.007	3.64	1.02	13.04	0.047
6-month mortality	4.51	1.85	11	0.001	3.35	1.31	8.58	0.012
12-month mortality	4.25	2	9	<0.001	3.02	1.35	6.72	0.007
Malnutrition according to MNA-SF								
3-month mortality	8.46	1.13	63.41	0.038	6.36	0.79	51.06	0.082
6-month mortality	7.38	1.74	31.29	0.007	5.71	1.28	25.36	0.022
12-month mortality	5.11	1.78	14.65	0.002	3.81	1.25	11.57	0.018

SGA: Subjective Global Assessment; MNA SF: Mini Nutritional Assessment Short Form; CI: Confidence interval.

## Data Availability

Not applicable.

## References

[1] Tebé C., del Río L.M., Casas L., Estrada M.D., Kotzeva A., Di Gregorio S., Espallargues M. (2011). Factores de riesgo de fracturas por fragilidad en una cohorte de mujeres españolas. Gac. Sanit..

[2] Rizzoli R., Biver E., Bonjour J.P., Coxam V., Goltzman D., Kanis J.A., Lappe J., Rejnmark L., Sahni S., Weaver C. (2018). Benefits and safety of dietary protein for bone health—An expert consensus paper endorsed by the European Society for Clinical and Economical Aspects of Osteopororosis, Osteoarthritis, and Musculoskeletal Diseases and by the International Osteoporosis Foundation. Osteoporos. Int..

[3] Nikkel L.E., Fox E.J., Black K.P., Davis C., Andersen L., Hollenbeak C.S. (2012). Impact of Comorbidities on Hospitalization Costs Following Hip Fracture. J. Bone Jt. Surg.-Am. Vol..

[4] Cummings S.R., Melton L.J. (2002). Epidemiology and outcomes of osteoporotic fractures. Lancet.

[5] Kanis J.A., Cooper C., Rizzoli R., Reginster J.Y. (2018). European guidance for the diagnosis and management of osteoporosis in postmenopausal women. Osteoporos. Int..

[6] Eisman J.A., Bogoch E.R., Dell R., Harrington J.T., McKinney R.E., McLellan A., Mitchell P.J., Silverman S., Singleton R., Siris E. (2012). Making the first fracture the last fracture: ASBMR task force report on secondary fracture prevention. J. Bone Miner. Res..

[7] Wu C.H., Tu S.T., Chang Y.F., Chan D.C., Chien J.T., Lin C.H., Singh S., Dasari M., Chen J.F., Tsai K.S. (2018). Fracture liaison services improve outcomes of patients with osteoporosis-related fractures: A systematic literature review and meta-analysis. Bone.

[8] González-Quevedo D., Pérez-Del-Río V., Moriel-Garceso D., Fernández-Arroyabe N., García-Meléndez G., Montañez-Ruiz M., Bravo-Bardají M., García-de-Quevedo D., Tamimi I. (2022). A 2-year follow-up of a novel Fracture Liaison Service: Can we reduce the mortality in elderly hip fracture patients? A prospective cohort study. Osteoporos. Int..

[9] Miu K.Y.D., Lam P.S. (2017). Effects of Nutritional Status on 6-Month Outcome of Hip Fractures in Elderly Patients. Ann. Rehabil. Med..

[10] Díaz de Bustamante M., Alarcón T., Menéndez-Colino R., Ramírez-Martín R., Otero Á., González-Montalvo J.I. (2018). Prevalence of malnutrition in a cohort of 509 patients with acute hip fracture: The importance of a comprehensive assessment. Eur. J. Clin. Nutr..

[11] Olofsson B., Stenvall M., Lundström M., Svensson O., Gustafson Y. (2007). Malnutrition in hip fracture patients: An intervention study. J. Clin. Nurs..

[12] Malafarina V., Reginster J.Y., Cabrerizo S., Bruyère O., Kanis J.A., Martinez J.A., Zulet M.A. (2018). Nutritional Status and Nutritional Treatment Are Related to Outcomes and Mortality in Older Adults with Hip Fracture. Nutrients.

[13] Pérez Durillo F.T., Ruiz López M.D., Bouzas P.R., Martín-Lagos A. (2010). Nutritional status in elderly patients with a hip fracture. Nutr. Hosp..

[14] Feng L., Chen W., Ping P., Ma T., Li Y., Xu L., Feng Z., Zhao Y., Fu S. (2022). Preoperative malnutrition as an independent risk factor for the postoperative mortality in elderly Chinese individuals undergoing hip surgery: A single-center observational study. Ther. Adv. Chronic Dis..

[15] Inoue T., Misu S., Tanaka T., Sakamoto H., Iwata K., Chuman Y., Ono R. (2017). Pre-fracture nutritional status is predictive of functional status at discharge during the acute phase with hip fracture patients: A multicenter prospective cohort study. Clin. Nutr..

[16] Inoue T., Maeda K., Nagano A., Shimizu A., Ueshima J., Murotani K., Sato K., Tsubaki A. (2020). Undernutrition, Sarcopenia, and Frailty in Fragility Hip Fracture: Advanced Strategies for Improving Clinical Outcomes. Nutrients.

[17] Muscaritoli M., Arends J., Bachmann P., Baracos V., Barthelemy N., Bertz H., Bozzetti F., Hütterer E., Isenring E., Kaasa S. (2021). ESPEN Guideline ESPEN practical guideline: Clinical Nutrition in cancer. Clin. Nutr..

[18] Avenell A., Smith T.O., Curtain J.P., Mak J.C., Myint P.K. (2016). Nutritional supplementation for hip fracture aftercare in older people. Cochrane Database Syst Rev..

[19] Bastow M.D., Rawlings J., Allison S.P. (1983). Benefits of supplementary tube feeding after fractured neck of femur: A randomised controlled trial. Br. Med. J. (Clin. Res. Ed.).

[20] Wyers C.E., Reijven P.L., Breedveld-Peters J.J., Denissen K.F., Schotanus M.G., van Dongen M.C., Eussen S.J., Heyligers I.C., van den Brandt P.A., Willems P.C. (2018). Efficacy of Nutritional Intervention in Elderly After Hip Fracture: A Multicenter Randomized Controlled Trial. J. Gerontol. A Biol. Sci. Med. Sci..

[21] Cederholm T., Jensen G.L., Correia M.I.T.D., Gonzalez M.C., Fukushima R., Higashiguchi T., Baptista G., Barazzoni R., Blaauw R., Coats A.J.S. (2019). GLIM criteria for the diagnosis of malnutrition—A consensus report from the global clinical nutrition community. J. Cachexia Sarcopenia Muscle.

[22] Kobayashi H., Inoue T., Ogawa M., Abe T., Tanaka T., Kakiuchi M. (2022). Malnutrition diagnosed by the Global Leadership Initiative on Malnutrition criteria as a predictor of gait ability in patients with hip fracture. Injury.

[23] Probert N., Lööw A., Akner G., Wretenberg P., Andersson G. (2020). A Comparison of Patients with Hip Fracture, Ten Years Apart: Morbidity, Malnutrition and Sarcopenia. J. Nutr. Health Aging.

[24] Millrose M., Schmidt W., Krickl J., Ittermann T., Ruether J., Bail H.J., Gesslein M. (2023). Influence of Malnutrition on Outcome after Hip Fractures in Older Patients. J. Pers. Med..

[25] Chavarro-Carvajal D.A., Dueñas-Orejuela M.F., Aruachan-Torres S.A., Caicedo Correa S.M., Segura Valencia A.I., Cano-Gutierrez C.A. (2022). Mortalidad al año y factores asociados en pacientes llevados a cirugía por fractura de cadera. Rev. Esp. Cir. Ortop. Traumatol..

[26] Charlson M.E., Pompei P., Ales K.L., MacKenzie C.R. (1987). A new method of classifying prognostic comorbidity in longitudinal studies: Development and validation. J. Chronic. Dis..

[27] Mahoney F.I., Barthel D. (1965). Functional Evaluation: The Barthel Index. Md. State Med. J..

[28] Holden M., Gill K., Magliozzi M., Nathan J., Piehl-Baker L. (1984). Clinical gait assessment in the neurologically impaired. Reliability and meaningfulness. Phys. Ther..

[29] Kaiser M.J., Bauer J.M., Ramsch C., Uter W., Guigoz Y., Cederholm T., Thomas D.R., Anthony P., Charlton K.E., Maggio M. (2009). Validation of the Mini Nutritional Assessment short-form (MNA-SF): A practical tool for identification of nutritional status. J. Nutr. Health Aging.

[30] Detsky A.S., Baker J.P., Johnston N., Whittaker S., Mendelson R.A., Jeejeebhoy K.N. (1987). What is subjective global assessment of nutritional status?. J. Parenter. Enter. Nutr..

[31] Torralvo F.J.S., Porras N., Fernández J.A., Torres F.G., Tapia M.J., Lima F., Soriguer F., Gonzalo M., Martínez G.R., Olveira G. (2018). Normative reference values for hand grip dynamometry in Spain. Association with lean mass. Nutr. Hosp..

[32] Cederholm T., Bosaeus I., Barazzoni R., Bauer J., Van Gossum A., Klek S., Muscaritoli M., Nyulasi I., Ockenga J., Schneider S.M. (2015). Diagnostic criteria for malnutrition—An ESPEN Consensus Statement. Clin. Nutr..

[33] Siri W.E. (1961). Body composition from fluid spaces and density: Analysis of methods. Nutrition.

[34] Durnin J.V., Womersley J. (1974). Body fat assessed from total body density and its estimation from skinfold thickness: Measurements on 481 men and women aged from 16 to 72 years. Br. J. Nutr..

[35] Barazzoni R., Jensen G.L., Correia M.I.T., Gonzalez M.C., Higashiguchi T., Shi H.P., Bischoff S.C., Boirie Y., Carrasco F., Cruz-Jentoft A. (2022). Guidance for assessment of the muscle mass phenotypic criterion for the Global Leadership Initiative on Malnutrition (GLIM) diagnosis of malnutrition. Clin. Nutr..

[36] Olveira G., Tapia M.J., Ocón J., Cabrejas-Gómez C., Ballesteros-Pomar M.D., Vidal-Casariego A., Arraiza-Irigoyen C., Olivares J., Conde-García M.C., García-Manzanares Á. (2013). The subjective global assessment predicts in-hospital mortality better than other nutrition-related risk indexes in noncritically ill inpatients who receive total parenteral nutrition in Spain (prospective multicenter study). J. Acad. Nutr. Diet..

[37] Contreras-Bolívar V., Sánchez-Torralvo F.J., Ruiz-Vico M., González-Almendros I., Barrios M., Padín S., Alba E., Olveira G. (2019). Glim criteria using hand grip strength adequately predict six-month mortality in cancer inpatients. Nutrients.

[38] Malafarina V., Malafarina C., Ugarte A.B., Martinez J.A., Goñi I.A., Zulet M.A. (2019). Factors Associated with Sarcopenia and 7-Year Mortality in Very Old Patients with Hip Fracture Admitted to Rehabilitation Units: A Pragmatic Study. Nutrients.

[39] Borges K., Artacho R., Jodar-Graus R., Molina-Montes E., Ruiz-López M.D. (2022). Calf Circumference, a Valuable Tool to Predict Sarcopenia in Older People Hospitalized with Hip Fracture. Nutrients.

[40] Ekinci O., Yanık S., Terzioğlu Bebitoğlu B., Yılmaz Akyüz E., Dokuyucu A., Erdem Ş. (2016). Effect of Calcium β-Hydroxy-β-Methylbutyrate (CaHMB), Vitamin D, and Protein Supplementation on Postoperative Immobilization in Malnourished Older Adult Patients With Hip Fracture. Nutr. Clin. Pract..

